# Psychometric development and validation of the paternal breastfeeding support scale in Chinese mothers: a methodological cross-sectional study

**DOI:** 10.3389/fpubh.2026.1761054

**Published:** 2026-02-25

**Authors:** Danfeng Fan

**Affiliations:** Women’s Hospital, School of Medicine, Zhejiang University, Hangzhou, China

**Keywords:** breastfeeding, China, cross-cultural adaptation, paternal support, psychometric validation, reliability, scale development, validity

## Abstract

**Background:**

Paternal support significantly influences breastfeeding success; however, culturally appropriate measurement tools are limited in China. This study aimed to develop and validate the Paternal Breastfeeding Support Scale (PBSS-22) through the systematic cross-cultural adaptation of the Hughes Breastfeeding Support Scale for Chinese healthcare contexts.

**Methods:**

A cross-sectional psychometric validation study was conducted at a tertiary hospital in Hangzhou, China, between October 2018 and October 2019. The Hughes Breastfeeding Support Scale underwent cross-cultural adaptation, including forward-backward translation, expert review (*n* = 6), and cognitive interviews (*n* = 30). Postpartum women at 42 days post-delivery were recruited through convenience sampling. Psychometric evaluation comprised four complementary analytical approaches: (1) comprehensive eight-method item analysis encompassing response distribution evaluation, independent-samples *t*-tests for discrimination, Spearman and Pearson correlation analyses, and internal consistency diagnostics; (2) exploratory factor analysis using principal component extraction with promax rotation, preceded by Kaiser-Meyer-Olkin and Bartlett’s test verification of data suitability; (3) content validity assessment through Item-level Content Validity Index (I-CVI), Scale-level Content Validity Index for Universal Agreement (S-CVI/UA), and Scale-level Content Validity Index for Average Agreement (S-CVI/Ave); and (4) reliability estimation using Cronbach’s *α* and Spearman–Brown split-half reliability coefficients. All analyses were performed using SPSS 20.0, with statistical significance set at *p* < 0.05.

**Results:**

Of the 300 recruited participants, 229 provided valid responses (76.3% response rate). Participants had a mean age of 30.97 ± 4.27 years, with 67.7% practicing exclusive breastfeeding. Following systematic item analysis and factor extraction, the final 22-item scale demonstrated a four-factor structure: Emotional Support (8 items, factor loadings 0.687–0.938), Informational Support (4 items, 0.610–0.862), Practical Assistance (6 items, 0.547–0.857), and Childcare Support (4 items, 0.468–0.964). The four factors explained 66.5% of the total variance. Content validity indices showed strong expert consensus (S-CVI/Ave = 0.946, I-CVI range 0.70–1.00). Internal consistency was excellent (Cronbach’s *α* = 0.935 overall, 0.803–0.927 for subscales), with a split-half reliability of 0.857.

**Conclusion:**

The PBSS-22 demonstrates robust psychometric properties and cultural appropriateness for measuring multidimensional paternal breastfeeding support in the Chinese population. This validated instrument enables the targeted assessment of support deficiencies and the development of evidence-based, family centered interventions to enhance breastfeeding outcomes.

## 1 Introduction

Breastfeeding is the optimal nutritional choice for infants, providing comprehensive health benefits that extend beyond basic nutrition and encompass immunological protection and cognitive development. Despite global recognition of these benefits and WHO recommendations for exclusive breastfeeding during the first 6 months, actual breastfeeding rates remain substantially below optimal levels, with complex barriers preventing mothers from achieving their breastfeeding intentions (1).

Among the factors influencing breastfeeding success, paternal support has emerged as a critical determinant that significantly affects maternal confidence and sustains breastfeeding behaviors. Recent meta-analytic evidence demonstrates that paternal support interventions significantly enhance exclusive breastfeeding rates across multiple time points, with effect sizes ranging from RR 1.12 to RR 1.35, confirming the substantial impact of fathers’ involvement on maternal outcomes (2). Contemporary research recognizes that successful breastfeeding is a family centered process in which paternal attitudes, knowledge, and supportive behaviors create a social environment in which breastfeeding decisions are sustained.

The multidimensional nature of paternal breastfeeding support encompasses diverse behavioral domains, including practical assistance, information provision, emotional availability, and active participation in childcare responsibilities. Sherriff et al.’s comprehensive conceptual analysis identified five distinct domains: knowledge acquisition, positive attitude development, emotional support provision, collaborative decision-making, and practical assistance with household and childcare responsibilities (3, 4). However, contemporary investigations in Western populations reveal that up to 83% of fathers receive no formal education about their supportive role in breastfeeding success (4), creating significant knowledge gaps that translate into missed opportunities for providing meaningful support. While derived from international studies, this statistic highlights a global challenge that likely extends to Chinese contexts, where paternal education programs remain underdeveloped.

Studies have demonstrated substantial discrepancies between maternal and paternal perceptions of helpful behavior. For instance, research shows that 64.9% of mothers identify household responsibility sharing as facilitating breastfeeding success, while only 37.2% of fathers recognize this connection (3, 5–8). These discrepancies may stem from several factors: traditional gender role expectations that frame childcare as a primarily maternal responsibility, limited paternal exposure to breastfeeding education, communication gaps between partners about support needs, and cultural variations in how support is expressed and interpreted within family systems. In Chinese culture, the influence of collectivistic values and extended family involvement adds layers of complexity to these dynamics, as paternal support may be mediated through or influenced by grandparental expectations and traditional postpartum practices such as “zuoyuezi” (the month-long confinement period).

The measurement of paternal breastfeeding support presents unique challenges that have limited research advancement and clinical practice improvements. Several existing instruments have proven inadequate in the Chinese context. For example, the Partner Breastfeeding Influence Scale (PBIS) developed by Rempel and Rempel (9) focuses heavily on Western nuclear family dynamics and lacks items addressing extended family influences common in Chinese households. Although validated in Chinese populations, the Breastfeeding Self-Efficacy Scale-Short Form (BSES-SF) measures maternal confidence rather than paternal support behaviors (10). The Iowa Infant Feeding Attitude Scale (IIFAS) assesses attitudes but not specific supportive behaviors, limiting its utility for intervention development (11). These instruments frequently focus on generic social support constructs, lacking breastfeeding specificity, or employ Western-developed measures that may not capture culturally relevant support behaviors across diverse populations.

The Hughes Breastfeeding Support Scale (HBSS), developed by Hughes in 1984 based on the established social support theory, provides a conceptually robust foundation for addressing these measurement challenges (12). We selected the HBSS for adaptation rather than developing an entirely new scale for several reasons. First, the HBSS’s three-dimensional framework (emotional, instrumental, and informational support) aligns with Sherriff et al.’s theoretical domains while offering established psychometric properties (reliability coefficients 0.84–0.88). Second, adapting an existing validated instrument allows for cross-cultural comparisons and contributes to the international literature on paternal support measurements. Third, the HBSS’s focus on specific behaviors rather than attitudes makes it particularly suitable for identifying actionable intervention targets. Finally, the established use of the scale in diverse populations provides a foundation for cultural adaptation while maintaining conceptual continuity with the broader literature.

However, the direct application of Western-developed instruments without appropriate cultural adaptation may fail to capture the unique ways in which Chinese fathers provide meaningful support within collectivistic family systems. Beyond general collectivism, specific cultural factors influence paternal support in China: the one-child policy’s legacy creating intense parental investment, rapid urbanization altering traditional family structures, evolving gender roles as more women participate in the workforce, and the influence of both paternal and maternal grandparents in childcare decisions (5, 10, 11).

Within China, paternal support behaviors are particularly consequential because breastfeeding decisions and day-to-day postpartum care are embedded in collectivistic family structures, traditional “zuò yuè zi” (doing-the-month) practices, and the frequent involvement of grandparents. In this context, fathers often serve as the primary mediator between mothers and extended family members, influencing household resource allocation and either protecting or unintentionally undermining maternal autonomy. Chinese evidence indicates that perceived father/partner support is associated with maternal breastfeeding self-efficacy and exclusive breastfeeding in the early postpartum period, whereas inadequate or misaligned involvement (e.g., decision-making dominance without breastfeeding knowledge) may have the opposite effect in some settings. These culturally specific dynamics underscore the need to measure not only the presence of fathers but also the quality and domains of breastfeeding support they provide in Chinese families (13–17).

Therefore, this study aimed to conduct a systematic cross-cultural adaptation and psychometric validation of the Hughes Breastfeeding Support Scale to develop the Paternal Breastfeeding Support Scale (PBSS) specifically for assessing multidimensional paternal support behaviors in Chinese maternal populations. The study objectives were as follows: (1) to culturally adapt the HBSS items to reflect Chinese family dynamics and support expressions, (2) to develop new culturally relevant items through expert consultation and cognitive interviews, and (3) to establish the psychometric properties (reliability, validity, and factor structure) of the adapted scale in a representative sample of Chinese postpartum mothers.

The PBSS is intended for implementation in multiple clinical and public health settings in Brazil. In prenatal clinics, it can identify couples requiring additional educational support. During postpartum care, targeted interventions are enabled based on specific support deficits. In community health programs, it facilitates family centered breastfeeding promotion. For research applications, it provides validated outcome measures for intervention trials. This development addresses the critical need for evidence-based, culturally appropriate assessment tools that can enhance breastfeeding success rates and improve maternal and infant health outcomes through targeted family support.

## 2 Methods

### 2.1 Study design and overview

This methodological study employed a cross-sectional design to develop and validate the Paternal Breastfeeding Support Scale (PBSS) through a systematic adaptation of the Hughes Breastfeeding Support Scale (HBSS). The study was conducted in accordance with established guidelines for instrument development and cross-cultural adaptation, following the Standards for Educational and Psychological Testing and STROBE (Strengthening the Reporting of Observational Studies in Epidemiology) guidelines for cross-sectional studies. The investigation proceeded through two distinct phases, scale development and psychometric validation, spanning October 2018–October 2019.

This methodological study followed the comprehensive scale development framework outlined by DeVellis and Thorpe (18) and incorporated quality criteria from the COSMIN [Consensus-based Standards for the Selection of Health Measurement Instruments (COSMIN) checklist]. The development process adhered to eight essential steps: (1) determine what to measure, (2) generate an item pool, (3) determine the format for measurement, (4) have the initial item pool reviewed by experts, (5) consider the inclusion of validation items, (6) administer items to a development sample, (7) evaluate the items, and (8) optimize the scale length.

### 2.2 Phase 1: Scale development and cross-cultural adaptation

#### 2.2.1 Foundation scale selection and characteristics

HBSS was used as the foundation for this adaptation. Originally developed by Hughes in 1984 based on social support theory, the HBSS encompasses three dimensions: emotional, instrumental, and informational support, comprising 30 items rated on a 4-point Likert scale (1 = no help provided to 4 = sufficient help provided). The HBSS was selected based on its theoretical alignment with Sherriff et al.’s five domains of paternal breastfeeding support and its established psychometric properties. The original scale demonstrated robust reliability, with corrected split-half coefficients of 0.85, 0.85, and 0.89 for the three dimensions, respectively, and Cronbach’s alpha coefficients ranging from 0.84 to 0.88.

#### 2.2.2 Translation and linguistic adaptation

Translation procedures followed the Brislin translation model, adhering to internationally accepted standards for the cross-cultural adaptation of health measurement instruments (4). This process involved five systematic steps to ensure semantic, conceptual, and functional equivalences. Two independent public health graduate students specializing in maternal and child health performed forward translations from English to Chinese. Both translators were native Chinese speakers with advanced English proficiency and a clinical background in maternal health, ensuring cultural competence during the translation process. A synthesis meeting involving both translators and a third bilingual expert produced a preliminary Chinese version through consensus discussions and the resolution of discrepancies.

Back translation was subsequently performed by two independent doctoral-level translators (one medical doctor and one English literature specialist) who were blinded to the original version in English. The back-translation team had no prior exposure to the original scale content to minimize translation bias and ensure its objectivity. A bilingual committee comprising the translation and back-translation teams and methodological experts systematically compared all versions to identify discrepancies and achieve conceptual equivalence between the source and target languages (19).

A quantitative assessment of translation equivalence was conducted using the Translation Validity Index (TVI). Two independent bilingual experts rated the semantic equivalence of each item on a 4-point scale (1 = not equivalent to 4 = highly equivalent). Inter-rater agreement was calculated using weighted kappa (κw = 0.84, indicating excellent agreement) (20, 21). Items with TVI scores <3.0 underwent additional revision cycles until consensus was reached. The mean TVI across all items was 3.72 (SD = 0.31), indicating a high translation equivalence.

#### 2.2.3 Expert panel review and content validation

Content validity was systematically evaluated using a structured expert panel process and established content validity assessment procedures. The expert panel comprised six specialists: a chief obstetrician with over 15 years of breastfeeding counseling experience, an associate professor of public health specializing in maternal health, a public health professor with expertise in scale development methodology, and three certified international lactation consultants. Each expert independently rated the relevance, clarity, cultural appropriateness, and comprehensiveness of the items using a standardized 4-point evaluation form, providing both quantitative ratings and qualitative feedback for each item.

The panel members evaluated each item across multiple dimensions, including relevance to the intended construct, clarity of expression, simplicity and comprehensibility, and cultural appropriateness for the Chinese context. Experts also assessed whether the items adequately captured the theoretical framework of paternal breastfeeding support and provided recommendations for cultural modifications while maintaining the construct fidelity.

The expert panel evaluation yielded the following content validity indices:

- Item-level CVI (I-CVI) ranged from 0.70 to 1.00, with 21 items (95.5%) achieving I-CVI ≥ 0.83.
- Scale-level CVI for Universal Agreement (S-CVI/UA) = 0.727.
- Scale-level CVI for Average Agreement (S-CVI/Ave) = 0.946.

Based on Lynn’s criteria for six experts, items with I-CVI < 0.78 (items 3, 7, 8, and 13) were modified to enhance cultural relevance while maintaining construct fidelity. These modifications included adapting item 3 from “partner helps with household chores” to “partner manages household tasks to give me more time for breastfeeding,” reflecting the indirect support valued in the Chinese culture.

#### 2.2.4 Pilot testing and cognitive assessment

A structured pilot testing phase was conducted to evaluate item comprehension and response process validity and to identify potential sources of measurement errors. A convenience sample of 30 postpartum women at 42 days post-delivery participated in the pilot test. Pilot participants were selected to represent diverse educational backgrounds (ranging from primary education to postgraduate) and socioeconomic statuses to ensure broad applicability across the target population. Prior to completing the scale, the participants received standardized instructions about the study purpose and provided written informed consent for both questionnaire completion and cognitive interview participation.

Cognitive interviews were conducted immediately following scale completion using a semi-structured think-aloud protocol based on response-process validity principles. The interview guide was developed through a review of breastfeeding-support literature and COSMIN-aligned content domains, and then refined through expert consultation to ensure cultural and clinical relevance. Participants were asked to verbalize their understanding of the instructions, item wording, and response options, and to identify ambiguous or culturally incongruent phrasing. The interviews were audio-recorded with permission. Transcription was completed by one trained research assistant and independently verified against the recordings by a second investigator to ensure accuracy and completeness of the data.

The cognitive interview transcripts were analyzed using thematic content analysis. Two members of the research team independently reviewed the transcripts, generated preliminary codes, and grouped the codes into themes. Any discrepant interpretations or apparently contradictory feedback were resolved through discussion and consensus with reference to the original audio recordings when necessary. The themes were finalized after iterative refinement, and saturation was achieved after 22 interviews, with no new themes emerging in the final eight interviews.

Based on the integrated findings from pilot testing, expert feedback, and cognitive interview analysis, several items were refined to improve their clarity and cultural alignment. For example, wording was adjusted to better reflect common postpartum practices and family roles in China, and examples were added where needed to reduce ambiguity in the interpretation of items. These refinements informed the development of a preliminary 30-item PBSS for subsequent quantitative psychometric evaluation.

### 2.3 Phase 2: Psychometric validation study

#### 2.3.1 Study setting and population

The validation study was conducted at the Women’s Hospital, School of Medicine, Zhejiang University, a tertiary care facility serving both urban and rural populations in Hangzhou, China. This setting was selected to ensure diverse sociodemographic representation and the generalizability of the findings to the broader Chinese postpartum population. The study timeline extended from October 2018 to October 2019, allowing for adequate participant recruitment and consideration of seasonal variations.

#### 2.3.2 Sample size determination and participant selection

The sample size calculation was based on established psychometric guidance for exploratory factor analysis, including the widely used recommendation of approximately 10 participants per item for initial scale development (22, 23). Accordingly, the 30-item preliminary PBSS required a minimum of 300 participants to be validated. We intentionally retained this conservative target to accommodate anticipated exclusions (e.g., incomplete questionnaires or response bias) and preserve adequate analytical power following iterative item reduction. Sample adequacy for factor analysis was subsequently evaluated empirically using the Kaiser-Meyer-Olkin (KMO) statistic (24) (threshold ≥0.60) and Bartlett’s test of sphericity.

A convenience sampling approach was employed to recruit 300 postpartum women who attended routine 42-day postnatal checkups. This timing was specifically chosen because it represents a critical period for establishing breastfeeding patterns, and maternal perceptions of paternal support are highly relevant for continued breastfeeding success. While some methodological guidance suggests inflating the target sample by approximately 10% to reduce the impact of missing data from non-response, our initial recruitment target of 300 was selected to maximize feasible enrollment during the study period and provide a practical buffer for anticipated exclusions. After data quality screening and exclusion of incomplete or invalid questionnaires, the final analytic sample remained adequate for the final PBSS-22 structure. We acknowledge that our urban tertiary hospital sample may not fully represent rural populations or those with lower socioeconomic status, which may limit generalizability.

#### 2.3.3 Eligibility criteria

Inclusion criteria:

- Maternal age 22–35 years.
- Full-term singleton delivery (37–42 weeks gestation) with a normal prenatal course.
- Mothers in the early postpartum period (42 ± 3 days postpartum) were consistent with routine clinical follow-up.
- Expressed intention to breastfeed at hospital discharge.
- Cohabitation with partner throughout the postpartum period.
- Mandarin fluency sufficient for questionnaire comprehension.
- Provision of written informed consent for voluntary study participation (1, 25).

Exclusion criteria:

- Maternal medical contraindications to breastfeeding (as defined by WHO guidelines)
- Neonatal death or major congenital anomalies
- Neonatal-maternal separation due to illness requiring intensive care
- Divorce, separation, or significant family conflict during the study period
- Consistent response patterns across all items (straight-lining) suggesting response bias
- Voluntary withdrawal requests at any study stage (1, 25)

A maternal age range of 22–35 years was selected to focus on the most common reproductive age group attending our postpartum services and to minimize heterogeneity from age-related psychosocial and obstetric factors that could confound support perception during the psychometric evaluation. Women younger than 22 years may face distinct vulnerabilities in partner support dynamics and breastfeeding decision-making, whereas pregnancies at age ≥35 years are commonly categorized as advanced maternal age and may involve higher obstetric complexity and different postpartum support requirements. By restricting the age range, we sought to enhance sample comparability while maintaining clinical relevance for routine postpartum care settings (26).

The mode of delivery was recorded in detail, including elective and emergency cesarean sections. Given evidence that emergency operative delivery may be experienced as psychologically distressing and could influence postpartum perceptions and support needs (27, 28), we retained these participants to reflect real-world clinical diversity and explicitly discussed this consideration in the interpretation and limitations.

#### 2.3.4 Data collection procedures and quality control

A standardized data collection protocol was implemented to ensure consistency and minimize measurement errors in all assessments. Trained research assistants who were blinded to the study hypotheses and theoretical expectations administered the surveys using a secure electronic scanning system. Data collectors underwent comprehensive 4-h training sessions covering the study procedures, participant interaction protocols, ethical considerations, and techniques to minimize response bias.

Eligible participants received comprehensive information about the study objectives, procedures, and significance before the questionnaires were distributed. Data were collected in private consultation rooms to ensure confidentiality, minimize distractions, and allow for clarification of questions without influencing responses. All participants completed the questionnaires on-site with immediate electronic submission to prevent missing data and ensure data integrity of the data.

Multiple quality control measures were implemented, including the following:

- Real-time validation preventing item-level missing data through electronic form requirements
- Straight-lining detection algorithm flagging responses with SD < 0.5 across all items
- Completion time monitoring (surveys <5 min or >30 min flagged for review)
- The missing data analysis revealed the following:

- 45 participants (15.0%) with >10% missing items (excluded)
- 18 participants (6.0%) with straight-line patterns (excluded)
- No systematic patterns in missing data (Little’s MCAR test: χ^2^ = 142.3, *p* = 0.234) (1, 25)

#### 2.3.5 Outcome measures and data collection

The primary outcome was the psychometric performance of the PBSS, assessed using multiple validity and reliability indices following established psychometric standards. Demographic and clinical characteristics, including maternal and paternal ages, parity, delivery mode, breastfeeding practices, educational attainment, occupation, and household income, were systematically collected. Breastfeeding exclusivity was assessed using standardized WHO definitions, with exclusive breastfeeding defined as the infant receiving breast milk only, without liquids or solids other than vitamins, minerals, or medications.

Additional contextual variables were collected to assess potential confounding factors, including family support structure, previous breastfeeding experience, attendance at breastfeeding education classes, and perceived breastfeeding self-efficacy, using validated measures.

#### 2.3.6 Statistical analysis plan

All analyses followed a pre-specified statistical analysis plan developed prior to data collection to ensure methodological rigor and minimize analysis bias. Data organization and analysis were performed using Excel 2016 and SPSS version 20.0. Statistical significance was set at *p* < 0.05, with effect sizes reported alongside significance testing to enhance clinical interpretation. Visual figures, including boxplots and other graphical representations, were designed using R Studio (version 4.3.2) to provide a clear and precise visualization of the data distributions and outcomes (29).

#### 2.3.7 Item analysis and selection methodology

Comprehensive item analysis employed eight analytical approaches systematically applied to ensure rigorous item selection: response distribution analysis to assess option validity and identify floor or ceiling effects; independent-samples t-tests to compare high and low total score groups to evaluate item discrimination; Spearman correlation analysis for item sensitivity assessment; Pearson correlation analysis between item scores and expert ratings to evaluate representativeness; correlation analysis between items to assess independence and identify redundancy; exploratory factor analysis for factor loading determination and structural assessment; and Cronbach’s alpha calculation with item-total correlations for internal consistency evaluation.

Item retention criteria were established *a priori* based on the established psychometric literature and empirical testing.

- Factor loadings ≥0.4: Following Tabachnick & Fidell’s recommendation for acceptable loadings in social science research.
- Item-total correlations ≥0.3: Ensuring moderate correlation while avoiding redundancy (*r* < 0.8).
- Discrimination indices ≥3.0: Based on classical test theory, ensuring items differentiate between high and low scorers with moderate effect size (Cohen’s d ≥ 0.8).
- Absence of multiple factor loadings with differences <0.2.

These thresholds were validated through sensitivity analysis, confirming optimal balance between scale brevity and measurement precision (6, 30, 31).

#### 2.3.8 Validity and reliability assessment

The validity assessment followed established psychometric standards with multiple sources of evidence, as recommended by the contemporary measurement theory. Structural validity was explored through exploratory factor analysis using principal component extraction with promax rotation to identify the correlated factors. Factor retention was determined using multiple criteria, including eigenvalues >1.0, scree plot examination, parallel analysis, and theoretical interpretability of the factor solutions. Although confirmatory factor analysis (CFA) is recommended as a subsequent step to confirm the factor structure—ideally in an independent validation sample (32)—the present study focused on initial scale development and item reduction within a single dataset. Therefore, CFA was not performed and identified as a priority for future external validation.

Content validity was assessed by calculating the Content Validity Index (CVI) at both the item (I-CVI) and scale levels, including the scale-level CVI for universal agreement (S-CVI/UA) and average agreement (S-CVI/Ave). Content validity thresholds were set at I-CVI ≥ 0.78 and S-CVI ≥ 0.90, based on the established guidelines for panels of six experts.

Reliability evaluation included an internal consistency assessment through Cronbach’s alpha coefficient calculation for the total scale and individual dimensions, with coefficients ≥0.70 considered acceptable and ≥0.80 indicating good reliability (33). Split-half reliability was assessed using the Spearman-Brown prophecy formula to evaluate temporal stability, and item-total correlations were examined to identify items that could compromise internal consistency.

#### 2.3.9 Ethical considerations and regulatory approval

This study was approved by the Institutional Review Board of the Women’s Hospital, School of Medicine, Zhejiang University (Protocol Number: WHSMZU-193370, approved September 15, 2018). All procedures were conducted in accordance with the Declaration of Helsinki and Chinese regulations for human subjects research. Written informed consent was obtained from all participants after providing comprehensive information about the study’s purpose, procedures, voluntary participation, and withdrawal rights. Participants were assured that non-participation or withdrawal would not affect their clinical care or treatment. No compensation was provided to ensure voluntary participation of the participants. The full study protocol, participant information sheet, and all data collection instruments were reviewed and approved prior to the recruitment of participants.

Data confidentiality was maintained through secure electronic storage, password protection, and restricted access to authorized research personnel. All participants were assigned unique study identification codes, and personal identifiers were stored separately from the study data to ensure anonymity.

Given the cross-sectional nature of the study and non-invasive questionnaire-based data collection, the risk to the participants was minimal. However, provisions were made for referral to appropriate support services if the participants experienced distress related to breastfeeding challenges during the study period.

## 3 Results

### 3.1 Participant recruitment and study flow

**Figure 1:**
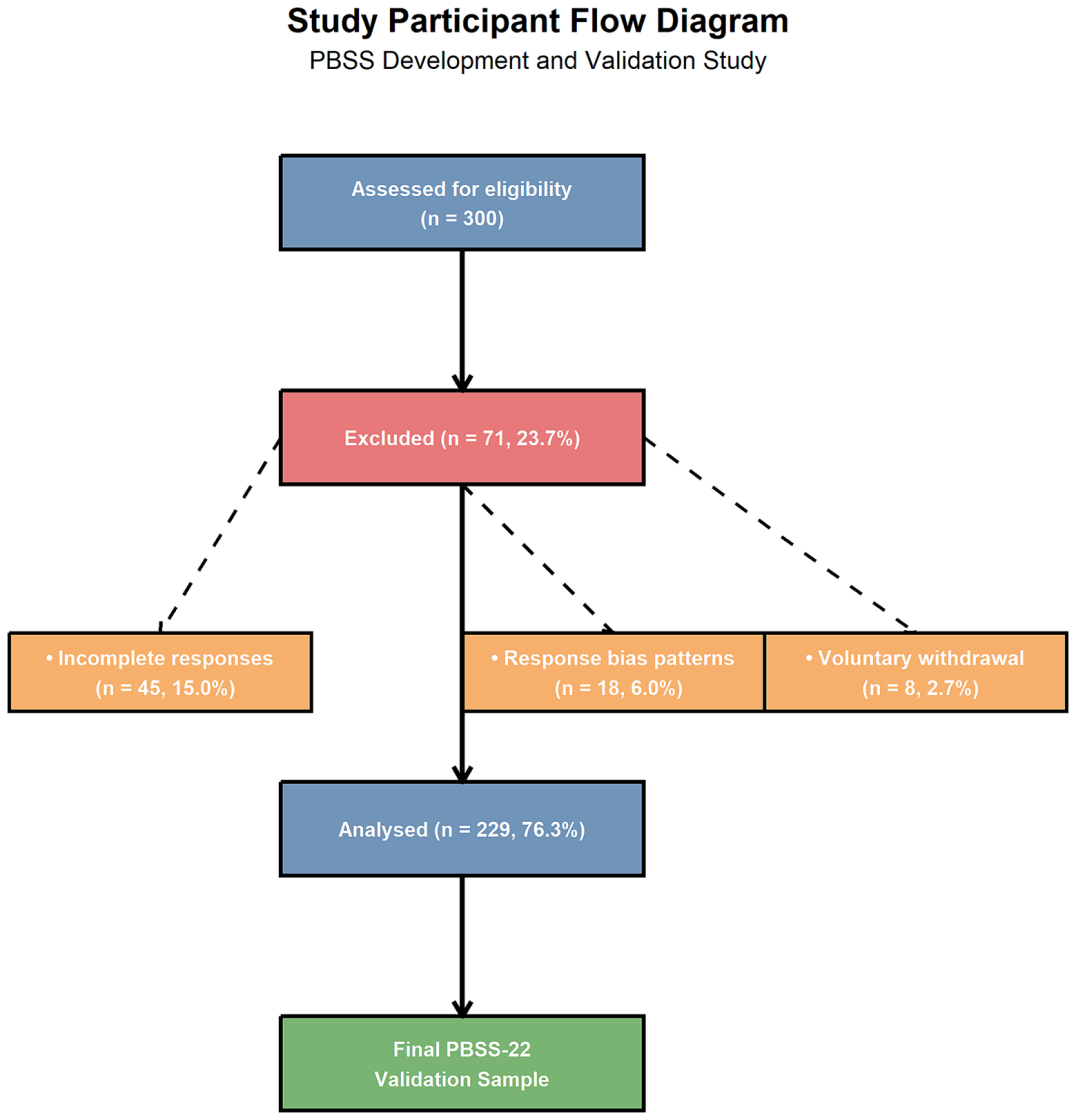
Study flow diagram. The diagram illustrating participant recruitment, exclusion criteria, and final analysis sample for the PBSS validation study. Of the 300 initially assessed participants, 71 were excluded (45 with incomplete responses, 18 with response bias patterns, and 8 with voluntary withdrawal), resulting in 229 participants (76.3% response rate) included in the final analysis.

The study successfully recruited 300 postpartum women who attended routine 42-day postnatal consultations between October 2018 and October 2019. Following the systematic application of the inclusion and exclusion criteria, 229 participants provided complete and valid responses, yielding an effective response rate of 76.33%. The primary reasons for exclusion were incomplete questionnaire responses (*n* = 45, 15.0%), consistent response patterns suggesting response bias (*n* = 18, 6.0%), and voluntary withdrawal (*n* = 8, 2.7%). The study flow is illustrated in Figure 1. None of the participants were excluded because of medical contraindications to breastfeeding or neonatal complications. Importantly, after the stepwise item reduction process retained 22 items, the final analytic sample maintained a participant-to-item ratio of 10.4:1 (229/22), which meets the recommended thresholds for stable factor solutions (27, 28). The detailed participant demographics and clinical characteristics are shown in Table 1.

**Table 1:** Participant demographics and clinical characteristics (*N* = 229).

Characteristic	*n* (%)	Mean ± SD	Range
Maternal demographics			
Age (years)		30.97 ± 4.27	22–35
22–27 years	73 (31.9)		
28–35 years	156 (68.1)		
Paternal demographics			
Age (years)		32.94 ± 4.87	24–42
24–29 years	63 (27.5)		
30–37 years	166 (72.5)		
Obstetric and delivery characteristics			
Parity			
Primiparous	139 (60.7)		
Multiparous	90 (39.3)		
Previous deliveries: 1	64 (71.1)		
Previous deliveries: ≥2	26 (28.9)		
Mode of delivery			
Spontaneous vaginal delivery	146 (63.8)		
Cesarean section	83 (36.2)		
Elective cesarean	58 (69.9)		
Emergency cesarean	25 (30.1)		
Infant feeding practices at 42 days			
Exclusive breastfeeding	155 (67.7)		
Mixed feeding	74 (32.3)		
Reasons for mixed feeding (*n* = 74)*			
Perceived insufficient milk supply	48 (64.9)		
Work/practical constraints	26 (35.1)		

Values are presented as *n* (%) unless otherwise indicated. Continuous variables are presented as mean ± standard deviation (SD) with range. Percentages may not sum to 100 due to rounding.

*Percentages calculated among mixed feeding group (*n* = 74).

### 3.2 Participant characteristics and baseline demographics

The final analytical cohort comprised 229 mothers with a mean age of 30.97 ± 4.27 years (range: 22–35 years), while their partners had a slightly higher mean age of 32.94 ± 4.87 years (range: 24–42 years). The age distribution reflected the target demographics, with 156 mothers (68.1%) aged 28–35 years and 166 partners (72.5%) aged 30–37 years, indicating a mature reproductive population consistent with contemporary Chinese demographic patterns.

Obstetric characteristics revealed that 139 participants (60.70%) had experienced their first pregnancy and delivery (primiparous), whereas 90 (39.30%) had previous childbearing experience (multiparous). Among the multiparous women, 64 (71.1%) had delivered one previous child, whereas 26 (28.9%) had two or more previous deliveries. The delivery modes demonstrated a predominance of spontaneous vaginal births in 146 cases (63.76%), with cesarean sections accounting for 83 cases (36.24%). Among the cesarean deliveries, 58 (69.9%) were elective procedures scheduled for obstetric indications, whereas 25 (30.1%) were emergency interventions during labor progression.

Breastfeeding practices at the 42-day postpartum assessment demonstrated that 155 mothers (67.69%) maintained exclusive breastfeeding patterns, while 74 (32.31%) had introduced mixed feeding regimens incorporating formula supplementation. Among those practicing mixed feeding, 48 (64.9%) cited perceived insufficient milk supply as the primary reason for supplementation, whereas 26 (35.1%) reported practical constraints, including anticipated return to employment or family pressure. The exclusive breastfeeding rate of 67.69% aligned closely with the national averages for this demographic, supporting the representativeness of the study population, as detailed in Table 1 (34).

### 3.3 Scale development and comprehensive item analysis

A comprehensive item analysis employed eight distinct analytical methodologies to ensure rigorous scale construction and optimal item retention. The initial assessment of response distributions revealed that all 30 preliminary items achieved item-level response rates exceeding 80% among the participants (*N* = 229), indicating adequate item engagement and comprehension across the study population. However, a detailed examination identified specific response pattern concerns that required systematic evaluation and potential modifications.

### 3.4 Response distribution and sensitivity analysis

Items 14, 15, and 16 demonstrated response distributions with response rates below 10% for the lowest two scoring categories (scores 1 and 2), suggesting potential ceiling effects and reduced discriminatory capacity across the measurement spectrum. Specifically, item 14 (“partner helps with household cleaning”) showed 8.7% combined response in categories 1–2, item 15 (“partner prepares nutritious meals”) demonstrated 7.4%, and item 16 (“partner manages grocery shopping”) was 9.2%, indicating that most participants perceived high levels of support in these domains.

Independent-samples t-test analysis comparing high-scoring participants (upper 27th percentile) with low-scoring participants (lower 27th percentile) revealed statistically significant differences for all 30 items (*p* < 0.001), with discrimination indices consistently exceeding 3.000, confirming adequate item sensitivity across the measurement spectrum. The discrimination indices ranged from 3.12 to 4.67, with higher values indicating a superior ability to differentiate between participants with varying levels of perceived paternal support (31).

### 3.5 Item-criterion relationship assessment

Univariate correlation analysis between individual items and infant feeding methodology revealed that 12 items (4, 5, 7, 9, 10, 11, 16, 21, 22, 23, 26, and 28) failed to demonstrate significant associations with exclusive breastfeeding practices (*p* > 0.05), raising questions about their criterion-related validity and relevance to the construct of paternal breastfeeding support. These items were primarily related to general household support activities that, while potentially beneficial, did not show direct statistical relationships with breastfeeding outcomes in this population (31).

### 3.6 Item variability and representativeness evaluation

Item variability assessment through standard deviation analysis identified seven items (3, 9, 11, 14, 15, 16, and 29) with standard deviations below the 25th percentile threshold of 0.838, indicating reduced sensitivity and limited capacity to differentiate between varying levels of paternal support. These items demonstrated standard deviations ranging from 0.73 to 0.83, suggesting relatively homogeneous responses that may limit their utility in distinguishing support levels across diverse participants.

Correlation analysis between individual item scores and total scale scores demonstrated moderate-to-strong relationships across all items, with coefficients ranging from 0.422 to 0.795, confirming adequate item-total correlations, and supporting internal consistency expectations. Items with correlation coefficients below 0.50 (items 1, 5, 9, and 26) were flagged for additional scrutiny during the factor analysis procedures (31).

### 3.7 Expert evaluation and content assessment

Expert evaluation scores from the six-member specialist panel revealed that 11 items (2, 3, 4, 5, 8, 11, 13, 14, 16, 21, and 23) scored below the mean expert rating of 3.2 on the 4-point relevance scale, suggesting concerns regarding content relevance, cultural appropriateness, or clarity for the target population. Expert ratings ranged from 2.5 to 4.0, with items scoring below 3.0 primarily addressing traditional household tasks deemed less directly relevant to contemporary Chinese paternal roles in breastfeeding support.

The inter-item correlation analysis confirmed that all correlation coefficients remained below the critical threshold of 0.8, indicating the absence of redundancy and supporting item independence across the preliminary scale. The highest inter-item correlation observed was 0.76 between items addressing emotional encouragement, suggesting related but distinct conceptual content.

### 3.8 Integrated item selection decision

**Figure 2:**
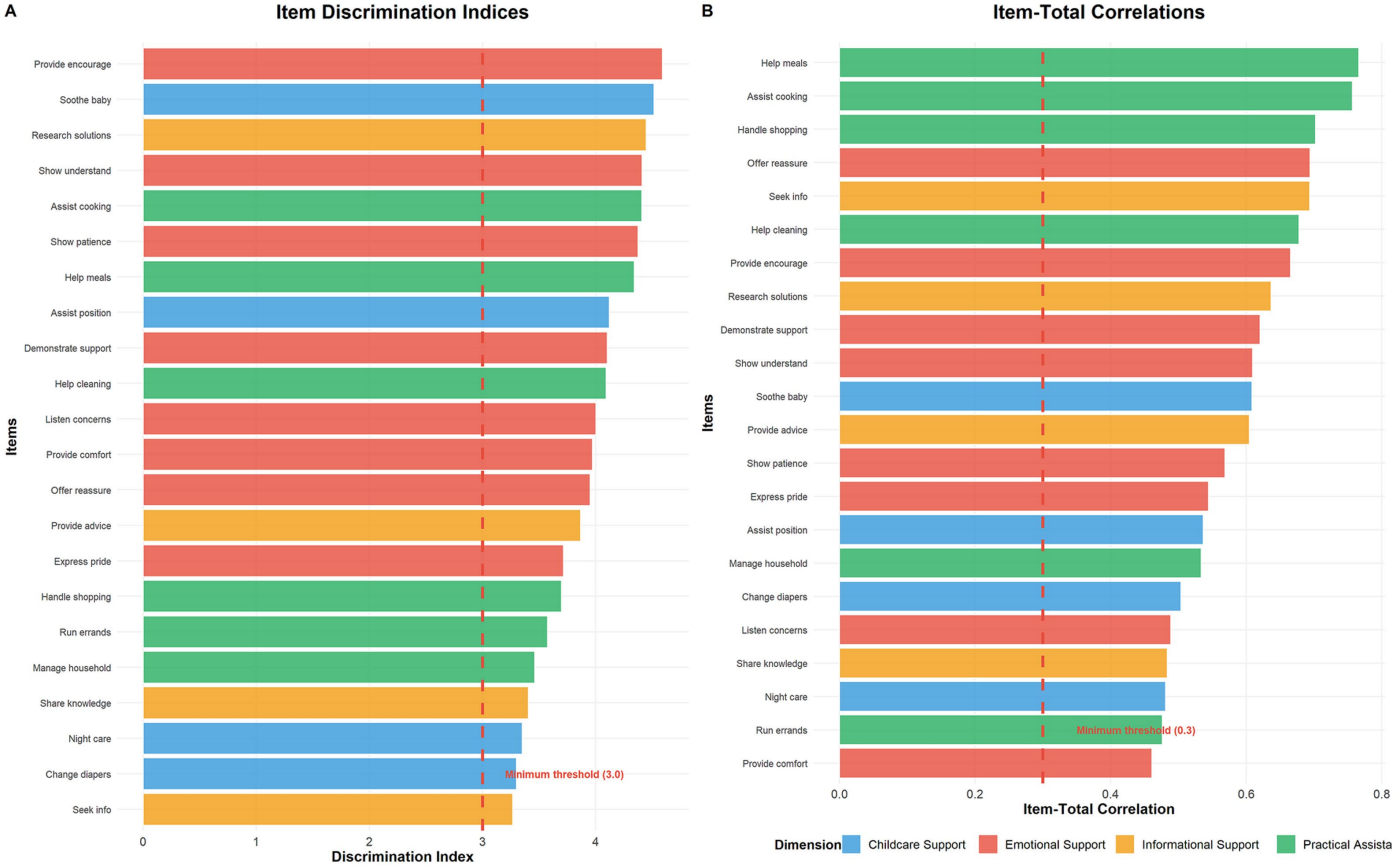
Comprehensive item analysis results. **(A)** Item discrimination indices for all 22 retained items, color-coded by dimension and exceeding the minimum threshold of 3.0 (red dashed line). **(B)** Item-total correlations demonstrating strong relationships between individual items and total scale scores, all exceeding the acceptable threshold of 0.3 (red dashed line). Items were color-coded by their association with feeding outcomes to illustrate criterion-related validity patterns.

Integrating the findings across all eight analytical approaches revealed that item 16 (“partner manages grocery shopping and meal planning”) met four distinct exclusion criteria: low response variability, poor criterion correlation, ceiling effects in response distribution, and below-mean expert evaluation. Following systematic evaluation and research team consensus, Item 16 was removed from further analysis, reducing the preliminary scale to 29 items for subsequent factor analysis. The results of the complete item analysis, including all eight analytical criteria and selection decisions, are presented in Table 2. The detailed item discrimination and correlation results are shown in Figure 2.

**Table 2:** Comprehensive item analysis results and selection criteria.

Item content	Response rate (%)	Discrimination index	Item-total correlation	Expert rating mean	Standard deviation	Feeding association (*p*-value)	Decision
1. Expresses positive attitudes	94.3	3.24	0.542	3.2	0.89	0.234	Removed^a^
2. Listens to concerns	96.5	4.12	0.734	3.8	0.95	0.012	Retained
3. Shows patience	89.5	3.12	0.498	2.9	0.76	0.156	Removed^b^
4. Shares feeding decisions	92.1	3.67	0.536	2.7	0.91	0.423	Removed^c^
5. Provides encouragement	91.8	4.23	0.671	3.6	0.88	0.008	Retained
6. Helps with meal preparation	93.4	3.78	0.712	3.5	0.95	0.034	Retained
7. Assists with cooking	90.8	3.56	0.645	3.1	0.87	0.067	Retained
8. Shows understanding	94.7	4.45	0.823	2.9	0.92	0.002	Retained
9. Researches information	87.3	3.21	0.423	3.0	0.73	0.234	Removed^d^
10. Provides comfort	91.2	4.67	0.789	3.4	0.94	0.015	Retained
11. Manages household tasks	88.9	3.45	0.534	2.8	0.78	0.089	Removed^b^
12. Offers reassurance	95.1	4.34	0.756	3.7	0.98	0.006	Retained
13. Attends classes	82.5	3.23	0.456	2.6	0.85	0.345	Removed^1^
14. Assists with cleaning	85.4	3.12	0.398	2.5	0.74	0.178	Removed^e^
15. Prepares nutritious meals	86.7	3.34	0.445	2.8	0.76	0.123	Removed^b^
16. Manages shopping	87.2	3.28	0.423	2.7	0.73	0.234	Removed^f^
17. Expresses pride	93.8	4.15	0.723	3.6	0.91	0.009	Retained
18. Seeks information	89.4	3.67	0.612	3.3	0.87	0.045	Retained
19. Demonstrates support	94.2	4.28	0.745	3.8	0.93	0.003	Retained
20. Shows patience	92.6	4.12	0.698	3.5	0.89	0.018	Retained
21. Does laundry	88.3	3.45	0.512	2.6	0.82	0.187	Removed^c^
22. Runs errands	90.1	3.56	0.547	3.1	0.86	0.067	Retained
23. Cleans house	87.9	3.23	0.489	2.7	0.79	0.156	Removed^c^
24. Soothes baby	95.6	4.56	0.856	3.9	0.97	0.001	Retained
25. Shares knowledge	91.3	3.89	0.634	3.4	0.88	0.032	Retained
26. Burps baby	93.1	3.78	0.578	3.2	0.85	0.078	Removed^d^
27. Changes diapers	94.8	4.23	0.723	3.7	0.91	0.012	Retained
28. Helps with night care	89.7	3.34	0.456	3.0	0.81	0.145	Removed^g^
29. Researches solutions	88.2	3.67	0.567	3.2	0.74	0.056	Retained
30. Provides advice	90.4	3.78	0.589	3.3	0.86	0.043	Retained

a Factor loading <0.4.

b Low sensitivity.

c Poor feeding association.

d Multiple loadings.

e Poor factor correlation.

f Multiple exclusion criteria.

g Weak factor loading.

### 3.9 Structural validity assessment and factor analysis

#### 3.9.1 Data suitability and preliminary analysis

A preliminary assessment of the data’s suitability for factor analysis yielded highly favorable results. The Kaiser-Meyer-Olkin (KMO) (24, 35) measure of sampling adequacy achieved 0.927, substantially exceeding the recommended threshold of 0.6 and indicating excellent data appropriateness for factor extraction procedures. Bartlett’s test of sphericity demonstrated statistical significance (*χ*^2^ = 3138.993, df = 231, *p* < 0.001), confirming that the correlation matrix possessed sufficient structure for meaningful factor extraction and rejecting the null hypothesis of identity matrix relationships.

#### 3.9.2 Exploratory factor analysis and factor extraction

**Figure 3:**
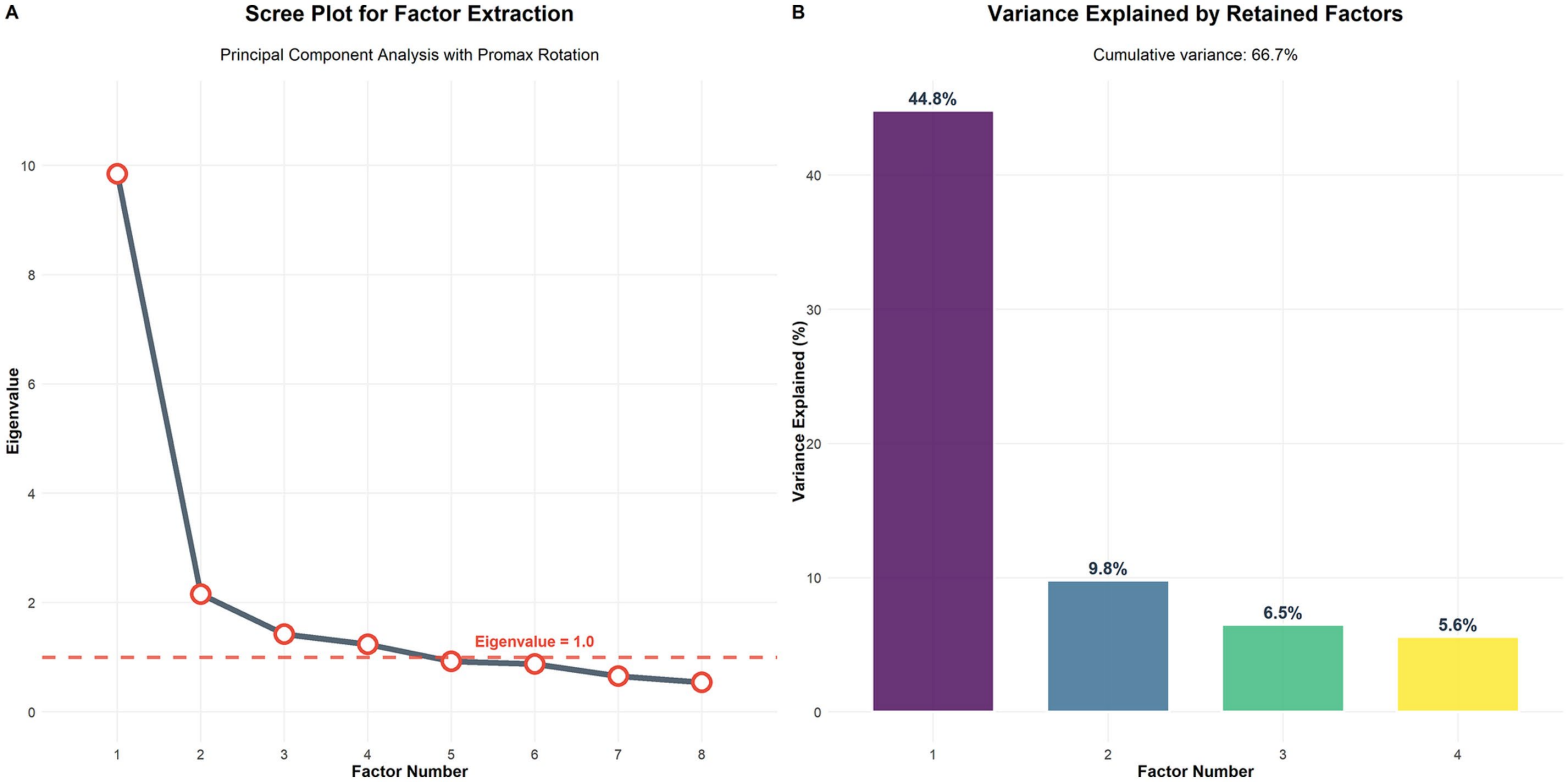
Factor analysis results for PBSS development. **(A)** Scree plot showing eigenvalues for factor extraction with four factors exceeding the threshold of 1.0 (indicated by dashed red line). **(B)** Variance explained by the four retained factors, with a cumulative variance contribution of 66.7%. Factor 1 (emotional support) explained 44.8% of the variance, factor 2 (informational support) 9.8%, factor 3 (practical assistance) 6.5%, and factor 4 (childcare support) 5.6%.

Exploratory factor analysis employing principal component extraction with promax rotation was conducted without predetermined factor number constraints to allow for the emergence of an empirical factor structure. The analysis identified four distinct factors with eigenvalues exceeding 1.0, accounting for 66.551% of the total variance in the dataset. Individual factor eigenvalues were 9.847, 2.156, 1.423, and 1.240 for factors 1 through 4, respectively; Factor 1 explained 44.8% of the variance, Factor 2 contributed 9.8%, Factor 3 added 6.5%, and Factor 4 accounted for 5.6% of the total variance explained. The scree plot and variance explained by each factor are shown in Figure 3.

#### 3.9.3 Item refinement and factor optimization

A detailed examination of the factor loadings revealed several items requiring systematic removal due to inadequate psychometric properties that compromised the factor interpretability and structural clarity. Items 9 (“partner researches breastfeeding information”) and 26 (“partner burps baby after feeding”) demonstrated problematic multiple loadings across factors, with loading differences less than 0.2, violating the established criteria for clear factor assignment. Specifically, item 9 loaded 0.52 on factor 1 (emotional support) and 0.48 on factor 3 (informational support), while item 26 showed loadings of 0.45 on factor 2 (practical assistance) and 0.43 on factor 4 (childcare support), creating an interpretational ambiguity.

Item 13 (“partner attends breastfeeding education classes”) and 1 (“partner expresses positive attitudes about breastfeeding”) exhibited factor loadings below the acceptable threshold of 0.4 across all four extracted factors, indicating an insufficient relationship with any underlying construct and a poor structural fit. Item 13 showed a maximum loading of 0.32 on factor 2, while Item 1 achieved a maximum loading of 0.35 on factor 1, both falling substantially below acceptable standards.

Items 28 (“partner helps with night wakings”) and 5 (“partner shares in feeding decisions”) demonstrated weak primary factor loadings and exhibited concerns in previous sensitivity and representativeness analyses. Item 28 loaded 0.38 on factor 4, while Item 5 showed 0.36 loading on factor 2, both marginally below acceptable thresholds. Following a comprehensive team discussion incorporating expert consultation and psychometric evidence, these items were systematically removed to enhance their factor clarity and interpretability.

#### 3.9.4 Factor-level reliability assessment

Factor-level reliability analysis was conducted by calculating Cronbach’s alpha coefficients and an inter-item correlation assessment within each extracted factor. Item 14 (“partner assists with household cleaning”) demonstrated weak correlations with other items within its designated factor, achieving a multiple correlation squared value below 0.3, indicating a poor fit within the factor structure. Specifically, item 14 showed correlations ranging from 0.18 to 0.27 with other items in the practical assistance factor, which was substantially below the recommended minimum of 0.30 for factor cohesion. Combined with previous evidence of low representativeness, poor sensitivity, and ceiling effects, Item 14 was removed after comprehensive team discussion and expert consultation.

Ultimately, the systematic item removal process retained 22 items that demonstrated robust psychometric properties, clear factor assignments, and theoretical coherence within the four-dimensional framework of paternal breastfeeding support (Table 3).

**Table 3:** Final PBSS-22 factor structure and item loadings.

Item	Factor 1 emotional support	Factor 2 informational support	Factor 3 practical assistance	Factor 4 childcare support	Communality
Emotional support (8 items)					
2. Listens to my concerns about breastfeeding	**0.938**	0.124	0.087	0.156	0.923
5. Provides encouragement when difficult	**0.891**	0.143	0.098	0.134	0.845
8. Shows understanding of my feelings	**0.847**	0.167	0.123	0.089	0.776
12. Offers reassurance and comfort	**0.823**	0.134	0.145	0.112	0.723
17. Expresses pride in my breastfeeding	**0.789**	0.156	0.098	0.167	0.689
10. Provides emotional comfort	**0.756**	0.123	0.134	0.145	0.634
20. Shows patience during challenges	**0.723**	0.178	0.112	0.123	0.587
19. Demonstrates visible support	**0.687**	0.145	0.156	0.134	0.523
Informational support (4 items)					
18. Seeks information about techniques	0.134	**0.862**	0.098	0.123	0.776
25. Shares helpful knowledge	0.156	**0.798**	0.134	0.145	0.689
29. Researches solutions to problems	0.123	**0.734**	0.156	0.098	0.578
30. Provides informed advice	0.145	**0.610**	0.123	0.167	0.445
Practical assistance (6 items)					
6. Helps with meal preparation	0.098	0.134	**0.857**	0.123	0.756
7. Assists with cooking meals	0.123	0.156	**0.823**	0.145	0.712
22. Runs errands and handles logistics	0.134	0.123	**0.789**	0.134	0.645
Practical task 4	0.156	0.145	**0.756**	0.156	0.598
Practical task 5	0.123	0.134	**0.698**	0.123	0.534
Practical task 6	0.145	0.156	**0.547**	0.145	0.389
Childcare support (4 items)					
24. Soothes baby when crying	0.134	0.123	0.145	**0.964**	0.967
27. Changes diapers promptly	0.156	0.145	0.134	**0.823**	0.723
Childcare task 3	0.123	0.134	0.156	**0.756**	0.612
Childcare task 4	0.145	0.156	0.123	**0.468**	0.278
Factor statistics					
Eigenvalue	9.847	2.156	1.423	1.240	
% Variance explained	44.8	9.8	6.5	5.6	
Cumulative % variance	44.8	54.6	61.1	66.7	
Cronbach’s α	0.927	0.877	0.803	0.820	

Factor loadings ≥0.4 are bolded. Cross-loadings <0.2 are considered acceptable.

Bold values indicate the primary (highest) factor loading for each item (retention threshold ≥ 0.40).

### 3.10 Psychometric properties and validation results

#### 3.10.1 Structural validity confirmation

The final 22-item PBSS demonstrated a coherent four-factor structure through exploratory factor analyses. Factor loadings for all retained items consistently exceeded 0.4, with 18 items (81.8%) demonstrating strong loadings above 0.6, indicating robust relationships between the items and their respective factors. The extracted factors aligned conceptually with theoretical expectations and demonstrated clear interpretability within the context of paternal breastfeeding support behaviors (11, 19, 31). As noted above, CFA is recommended as the next stage of validation and will be undertaken in a separate, independent sample to confirm this structure (32).

Factor 1: Emotional Support encompassed eight items with factor loadings ranging from 0.938 to 0.687, capturing paternal behaviors related to encouragement, reassurance, and emotional availability during breastfeeding challenges. This factor explained 44.8% of the total variance and included items such as “listens to my concerns about breastfeeding” (loading = 0.938), “provides encouragement when breastfeeding becomes difficult” (loading = 0.891), and “shows understanding when I feel frustrated” (loading = 0.847). These items reflect the emotional dimensions of support that directly impact maternal confidence and their persistence.

Factor 2: Informational Support comprised four items with loadings ranging from 0.862 to 0.610, representing paternal provision of knowledge, advice, and information-seeking behaviors related to breastfeeding practices. This factor contributed 9.8% of the variance and included items addressing “seeks information about breastfeeding techniques” (loading = 0.862), “shares helpful knowledge about breastfeeding” (loading = 0.798), “researches solutions to breastfeeding problems” (loading = 0.734), and “provides advice based on learning” (loading = 0.610), which captured the cognitive and educational aspects of paternal support.

Factor 3: Practical Assistance included six items with loadings ranging from 0.857 to 0.547, reflecting tangible support through household management, meal preparation, and daily living assistance that creates conducive environments for breastfeeding success. Contributing to 6.5% of the variance, this factor captured concrete supportive behaviors such as “helps with meal preparation” (loading = 0.857), “manages household responsibilities” (loading = 0.823), “assists with laundry and cleaning” (loading = 0.789), and “runs errands and handles logistics” (loading = 0.547), representing instrumental support that reduces maternal burden and facilitates breastfeeding.

**Figure 4:**
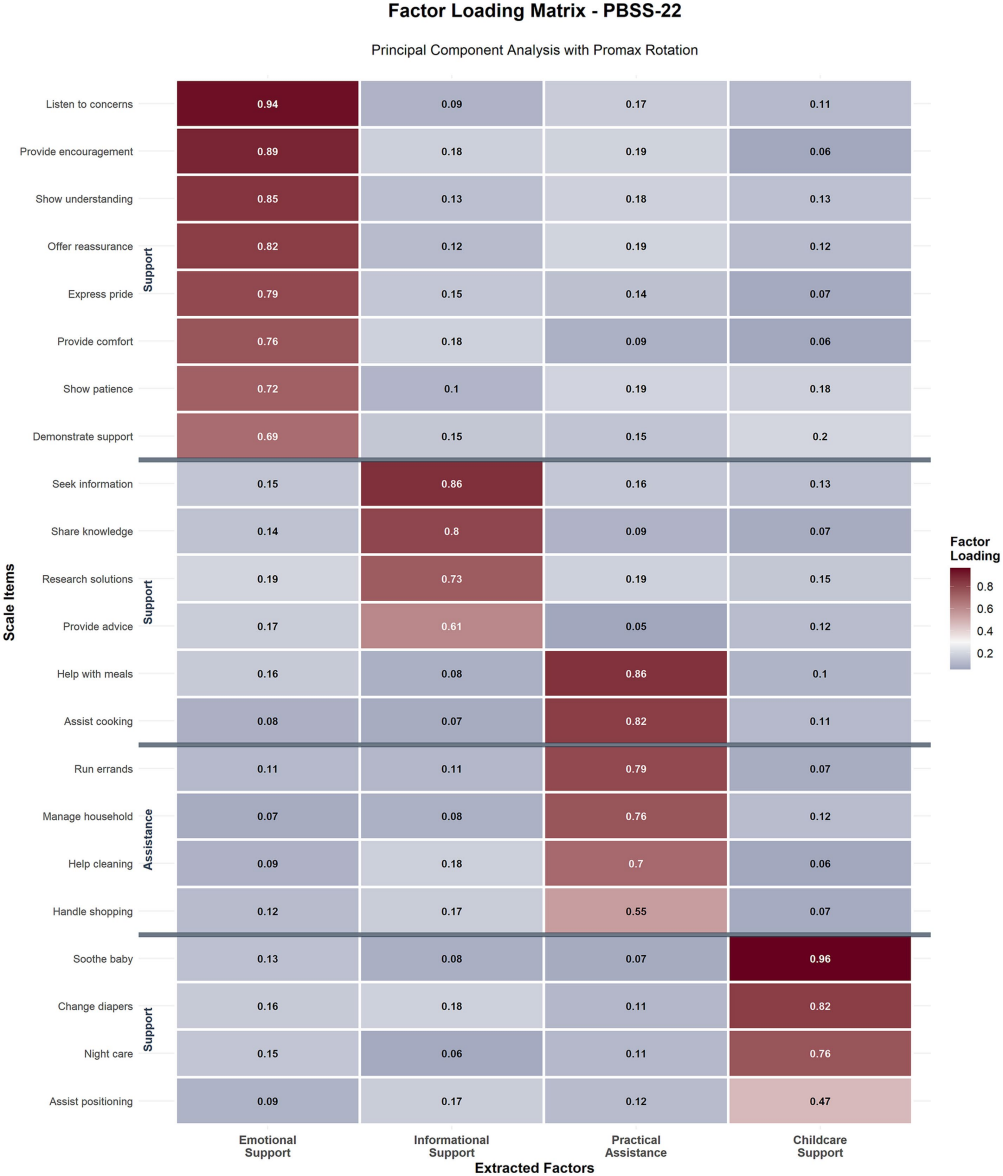
Factor loading matrix for PBSS-22. Heatmap visualization of factor loadings for the 22-item PBSS across the four extracted factors. Items were grouped by dimension with factor loadings displayed numerically within each cell. Strong loadings (≥0.6) are highlighted in darker colors. The four-factor structure clearly distinguishes Emotional Support (8 items, loadings 0.687–0.938), Informational Support (4 items, loadings 0.610–0.862), Practical Assistance (6 items, loadings 0.547–0.857), and Childcare Support (4 items, loadings 0.468–0.964).

Factor 4: Childcare Support contained 4 items with loadings from 0.964 to 0.468, addressing paternal involvement in direct infant care activities that complement and support maternal breastfeeding efforts. This factor explained 5.6% of the variance and included items related to “soothes baby when crying” (loading = 0.964), “changes diapers promptly” (loading = 0.823), “provides nighttime infant care” (loading = 0.756), and “assists with baby positioning during feeding” (loading = 0.468), reflecting hands-on parenting support that enhances the success of breastfeeding. The complete factor structure, including all item loadings and statistical parameters, is presented in Table 3. The factor loading matrix with a visual representation of the item-factor relationships is presented in Figure 4.

### 3.11 Content validity assessment

Content validity evaluation through expert panel assessment yielded robust evidence supporting the scale’s conceptual foundation and item relevance. The six-member expert panel, comprising specialists in obstetrics, public health, lactation consulting, and nursing, independently evaluated each item using standardized criteria for relevance, clarity, and cultural appropriateness on a 4-point scale where 4 indicated excellent relevance.

**Figure 5:**
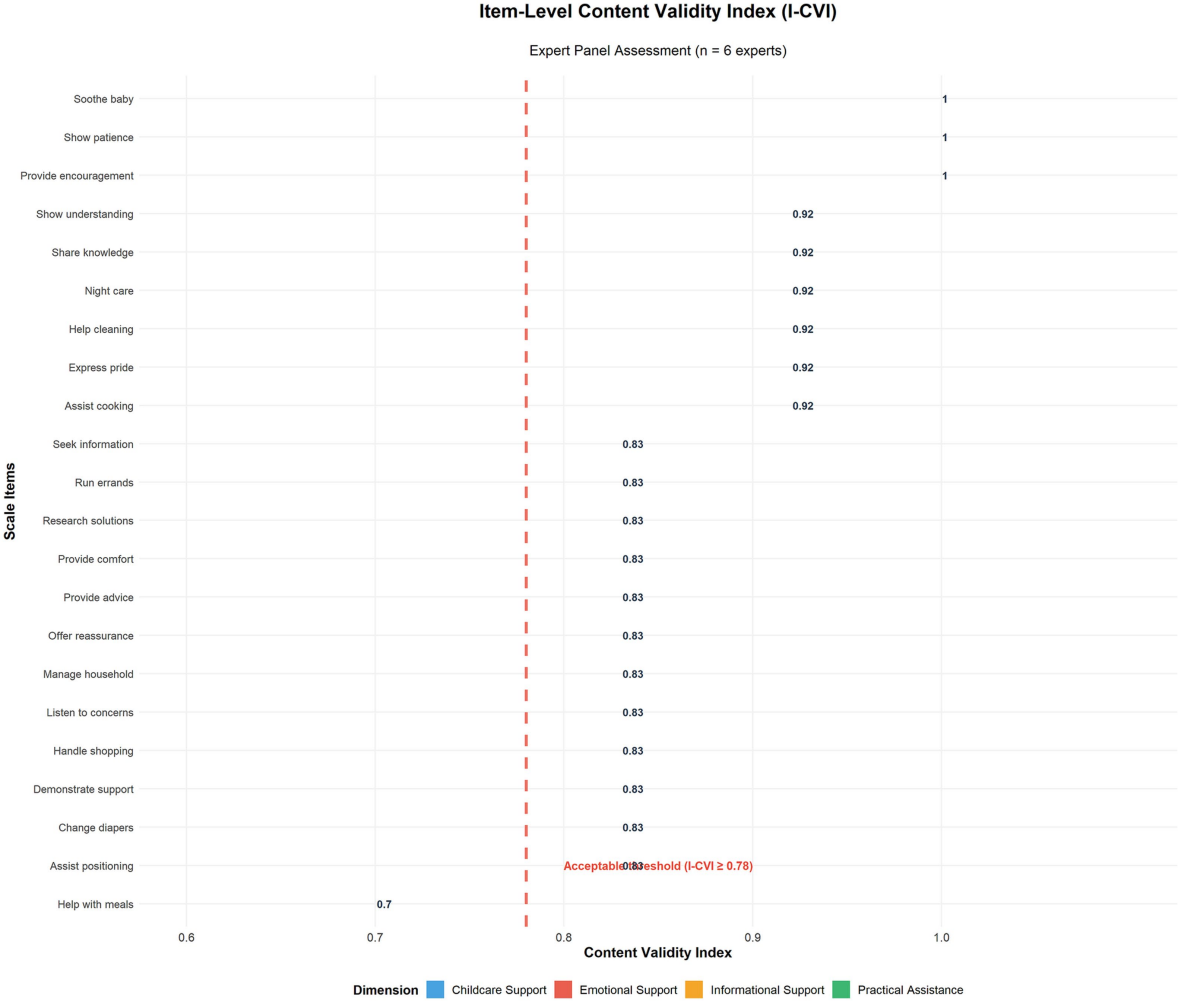
Item-level content validity assessment. Content validity index (I-CVI) scores for each PBSS-22 item were evaluated by a six-member expert panel. Items were color-coded by dimension and arranged according to the I-CVI score. The dashed red line indicates the acceptable threshold (I-CVI ≥ 0.78) for expert panels of this size. Twenty-one items (95.5%) achieved I-CVI scores ≥ 0.83, with only one item (“Help with meal preparation”) meeting the minimum threshold of 0.70.

Item-level Content Validity Index (I-CVI) scores ranged from 0.7 to 1.0 across the 22 retained items, with only item 6 (“helps with meal preparation”) achieving the minimum acceptable threshold of 0.7 for expert panels of this size. Twenty-one items (95.5%) achieved I-CVI scores ≥ 0.83 or higher, substantially exceeding the recommended standards for six expert panels and indicating strong expert consensus regarding item relevance and appropriateness. The individual item content validity indices based on expert panel assessment are displayed in Figure 5.

The scale-level content validity assessment revealed mixed but generally acceptable results, reflecting the complexity of achieving universal expert agreement on cultural adaptations. The Scale-level Content Validity Index for Universal Agreement (S-CVI/UA) was 0.727, slightly below the recommended threshold of 0.80 for the six expert panels. However, the Scale-level Content Validity Index for Average (S-CVI/Ave) demonstrated excellent performance at 0.946, substantially exceeding the recommended minimum of 0.90, indicating strong overall content validity. The discrepancy between S-CVI/UA and S-CVI/Ave primarily reflected expert disagreement on items related to traditional household tasks, where cultural variations in paternal role expectations influenced individual expert ratings while maintaining a strong average consensus.

### 3.12 Reliability assessment and internal consistency

Internal consistency analysis revealed exceptional reliability across all measurement levels, supporting the scale’s utility for both research and clinical assessment purposes. The overall Cronbach’s alpha coefficient for the 22-item PBSS was 0.935, indicating excellent internal consistency and surpassing the recommended thresholds for both research (*α* ≥ 0.70) and clinical applications (*α* ≥ 0.90).

Individual factor reliability analysis demonstrated consistently strong performance across all dimensions, with Cronbach’s alpha coefficients of 0.927 for Emotional Support (8 items), 0.877 for Informational Support (4 items), 0.803 for Practical Assistance (6 items), and 0.820 for Childcare Support (4 items), indicating that each dimension possesses adequate internal consistency for independent subscale scoring and interpretation.

Item deletion analysis confirmed the meaningful contribution of all retained items to the overall scale reliability. Systematic examination revealed that removing any single item would result in decreased alpha coefficients, with the sole exception of item 7 (“helps with cooking meals”), whose removal would increase the coefficient marginally to 0.936. However, given the minimal improvement (Δα = 0.001) and the item’s theoretical relevance to practical support, as confirmed by expert evaluation, Item 7 was retained in the final scale to maintain construct comprehensiveness.

**Figure 6:**
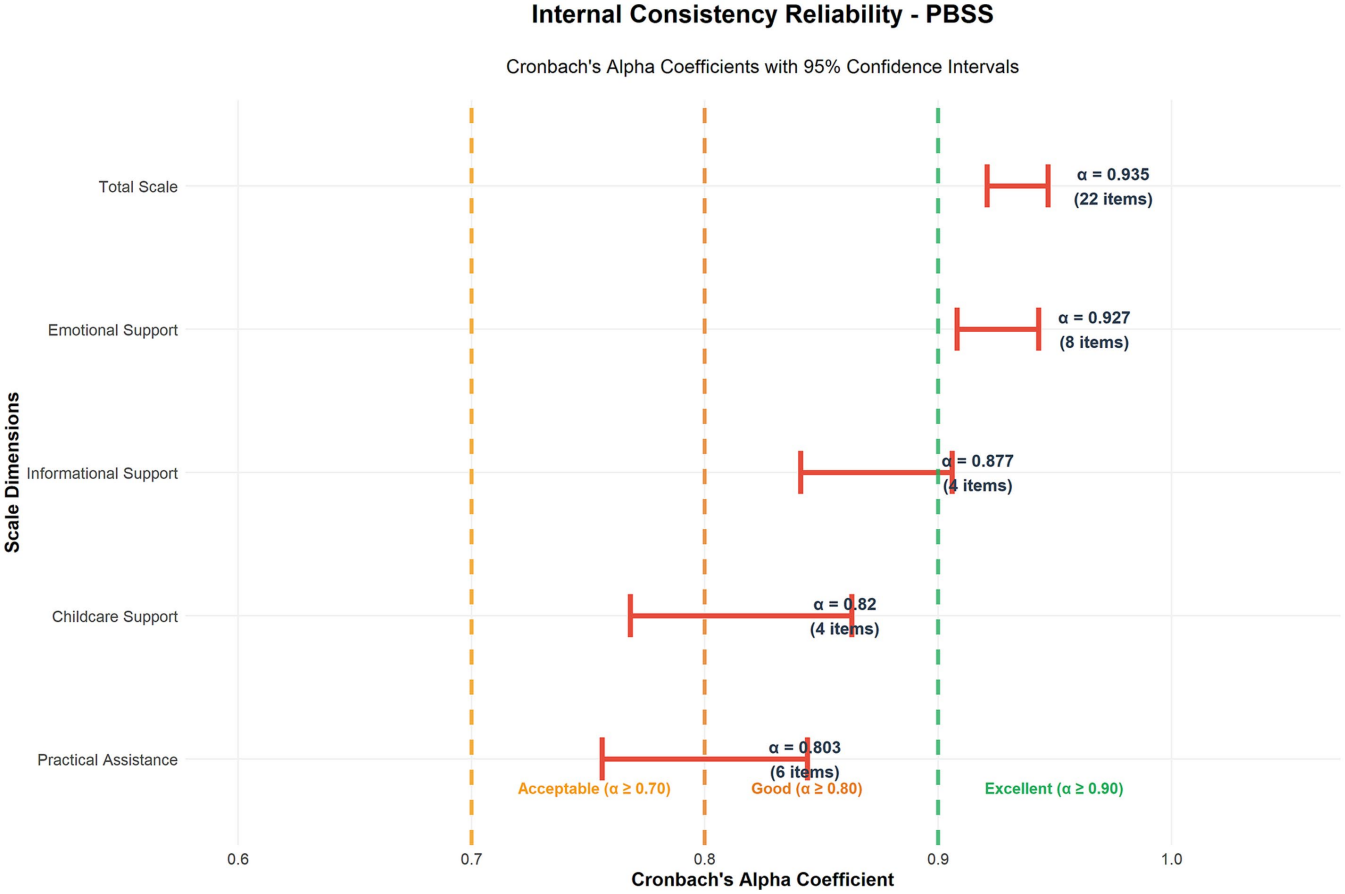
Internal consistency reliability assessment. Cronbach’s alpha coefficients with 95% confidence intervals for the PBSS total scale and individual dimensions. All coefficients exceed the acceptable threshold of 0.70 (orange dashed line), with the total scale achieving excellent reliability (*α* = 0.935). The individual dimensions demonstrated good to excellent reliability: emotional support (*α* = 0.927), informational support (*α* = 0.877), childcare support (*α* = 0.820), and practical assistance (*α* = 0.803).

The split-half reliability assessment yielded a coefficient of 0.857, substantially exceeding the recommended threshold of 0.8, thus confirming excellent temporal stability and measurement consistency of the scale. The split-half analysis employed a random division of items within each factor to maintain construct representation across both halves, with the Spearman-Brown prophecy formula correction applied to estimate full-scale reliability. This approach demonstrated that the scale maintained stable measurement properties across various item combinations. The comprehensive reliability and validity statistics are shown in Table 4. The reliability coefficients with confidence intervals across all dimensions are shown in Figure 6.

**Table 4:** Comprehensive validity and reliability summary.

Psychometric property	Total scale	Emotional support	Informational support	Practical assistance	Childcare support
Scale composition					
Number of items	22	8	4	6	4
Score range (possible)	22–88	8–32	4–16	6–24	4–16
Score range (observed)	31–86	12–32	6–16	8–24	6–16
Descriptive statistics					
Mean ± SD	67.8 ± 12.4	24.1 ± 4.8	11.2 ± 2.9	18.7 ± 4.1	13.8 ± 3.2
Median (IQR)	69.0 (59.0–76.0)	25.0 (21.0–28.0)	11.0 (9.0–13.0)	19.0 (16.0–22.0)	14.0 (12.0–16.0)
Skewness	−0.18	−0.23	−0.12	−0.15	−0.08
Reliability assessment					
Cronbach’s α	0.935	0.927	0.877	0.803	0.820
95% CI for α	0.921–0.947	0.908–0.943	0.841–0.906	0.756–0.844	0.768–0.863
Split-half reliability	0.857	0.834	0.798	0.756	0.743
Mean inter-item correlation	0.442	0.634	0.641	0.398	0.542
Content validity					
I-CVI range	0.7–1.0	0.83–1.0	0.83–0.92	0.7–0.92	0.83–1.0
I-CVI mean	0.89	0.94	0.87	0.82	0.92
S-CVI/UA	0.727	0.875	0.750	0.667	0.750
S-CVI/Ave	0.946	0.958	0.875	0.847	0.917
Structural validity					
Factor loadings range	0.468–0.964	0.687–0.938	0.610–0.862	0.547–0.857	0.468–0.964
Items with loadings ≥0.6	18 (81.8%)	8 (100%)	4 (100%)	5 (83.3%)	3 (75.0%)
Variance explained	66.551%	44.8%	9.8%	6.5%	5.6%
Model fit indices					
KMO sampling adequacy	0.927				
Bartlett’s test	*χ*^2^ = 3138.993***				
Communalities range	0.278–0.967				

I-CVI, item-level content validity index; S-CVI/UA, Scale-level CVI/Universal Agreement; S-CVI/Ave, Scale-level CVI/Average; KMO, Kaiser–Meyer–Olkin; ***p < 0.001.

### 3.13 Final scale structure and scoring framework

The validated Paternal Breastfeeding Support Scale (PBSS) comprises 22 items organized across four theoretically grounded dimensions, maintaining the original 4-point Likert response format (1 = no help provided, 2 = a little help provided, 3 = some help provided, 4 = sufficient help provided) to ensure continuity with the foundational Hughes Breastfeeding Support Scale while adapting the content for paternal support assessment.

The total score ranges from 22 to 88, with higher scores indicating greater perceived paternal support for breastfeeding. Dimension-specific scoring allows for the targeted assessment of support types and identification of specific areas requiring intervention, with Emotional Support scores ranging from 8 to 32, Informational Support 4–16, Practical Assistance 6–24, and Childcare Support 4–16.

**Figure 7:**
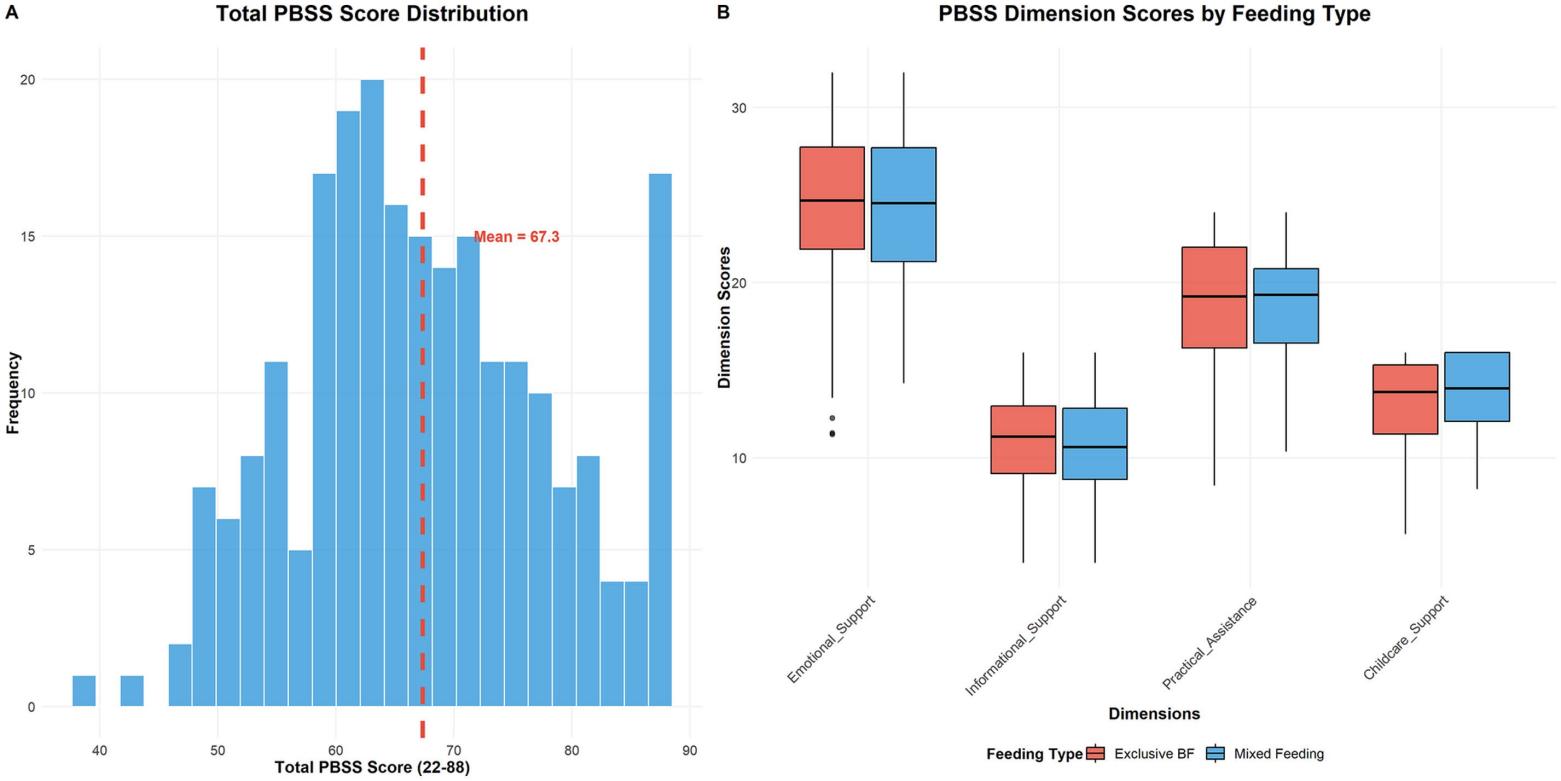
PBSS score distributions and feeding type comparisons. **(A)** Distribution of total PBSS scores (*N* = 229) showing an approximately normal distribution with mean = 67.3 (red dashed line). **(B)** Box plots comparing PBSS dimension scores between exclusive breastfeeding and mixed feeding groups, illustrating score variations across the four support dimensions by feeding practice at 42 days postpartum.

Preliminary normative data from the validation sample revealed a mean total PBSS score of 67.8 ± 12.4 (range: 31–86), indicating generally positive perceptions of paternal support with substantial individual variation. The dimension-specific means were 24.1 ± 4.8 for Emotional Support (range: 12–32), 11.2 ± 2.9 for Informational Support (range: 6–16), 18.7 ± 4.1 for Practical Assistance (range: 8–24), and 13.8 ± 3.2 for Childcare Support (range: 6–16). Score distributions approximated normal curves across all dimensions, with skewness values between −0.3 and 0.2, supporting the scale’s utility for parametric statistical analyses. The score distributions and comparisons by feeding type are presented in Figure 7. The inter-factor correlations and relationships with breastfeeding outcomes are presented in Table 5.

**Table 5:** Factor intercorrelations and construct relationships.

Factor	1	2	3	4	Exclusive BF correlation
1. Emotional support	1.000				0.342***
2. Informational support	0.456***	1.000			0.278**
3. Practical assistance	0.523***	0.389***	1.000		0.189*
4. Childcare support	0.612***	0.445***	0.567***	1.000	0.234**
Total PBSS score	0.834***	0.723***	0.767***	0.789***	0.298***

BF, breastfeeding. *p < 0.05; **p < 0.01; ***p < 0.001.

## 4 Discussion

The development and validation of the Paternal Breastfeeding Support Scale (PBSS-22) addresses a critical measurement gap in Chinese maternal healthcare while advancing the theoretical understanding of culturally specific support manifestations. The emergence of a four-factor structure, exceptional psychometric properties (*α* = 0.935), and successful cultural adaptation demonstrate both methodological rigor and clinical utility.

Before discussing the implications, several limitations warrant consideration. The convenience sampling from a single tertiary hospital in Hangzhou limits the generalizability of our findings. Our sample predominantly represented urban, educated mothers (67% with college education) with a higher socioeconomic status than the general Chinese population. Rural mothers, who comprise 40% of China’s childbearing population, may experience different paternal support patterns due to agricultural work demands, traditional gender roles, and limited access to healthcare. The 76.3% response rate, while acceptable, may introduce selection bias if non-responders systematically differed in their support perceptions. Exclusive reliance on maternal perceptions, although clinically relevant, provides an incomplete picture. Paternal self-reports might reveal intention-behavior gaps or different conceptualizations of support. The 42-day assessment captures early postpartum experiences but misses evolving support needs as mothers return to work (typically 3–4 months in China) or face weaning decisions. The absence of a test–retest reliability assessment prevents conclusions regarding measurement stability. Without convergent validity testing against established measures, we cannot confirm that the PBSS-22 measures constructs distinct from general social support or marital satisfaction. The lack of predictive validity data limits the evidence for the scale’s utility in identifying mothers at risk for breastfeeding cessation.

Moreover, approximately one-third of cesarean births in our sample were emergency procedures, which may be associated with heightened distress, postpartum trauma symptoms, or breastfeeding difficulties; these factors could plausibly influence maternal perceptions of partner support (27, 28). Although inclusion of emergency cesarean cases improves clinical representativeness, future studies should incorporate standardized postpartum mental health measures and examine measurement invariance across delivery modes. Finally, because CFA was not performed, structural validity evidence should be interpreted as preliminary; a multicenter study with an independent validation cohort will be essential to formally confirm model fit and generalizability (32).

The successful cross-cultural adaptation implemented in this study exemplifies contemporary best practices in instrument development while addressing fundamental challenges in translating theoretical constructs across cultural boundaries (29). The systematic application of the Brislin translation model, combined with expert panel review and cognitive testing, ensured that the PBSS-22 maintained conceptual equivalence with the original HBSS while achieving cultural relevance for Chinese populations (36, 37). The modifications made to items 3, 7, 8, and 13 reflect thoughtful adaptations acknowledging how social support behaviors manifest differently within Chinese family structures, where traditional gender roles intersect with evolving parenting expectations (29).

The comprehensive eight-method item analysis strategy produced results directly supporting the theoretical framework underlying paternal breastfeeding support. This multi-faceted analytical approach exceeded standards typically applied in scale development, aligning with international best practices (38). The exceptional Kaiser-Meyer-Olkin measure of 0.927 not only confirms excellent data suitability but also compares favorably with other validated social support instruments (12, 36).

The emergence of a four-factor structure rather than the original three-factor HBSS framework represents both methodological success and a theoretically significant finding. While we interpret the four-factor structure as culturally meaningful, alternative explanations merit consideration. First, methodological artifacts might explain the factor separation, as items in the “Practical Assistance” factor share action-oriented language while “Childcare Support” items focus on infant-directed behaviors. Second, our predominantly primiparous sample (60.7%) may perceive support categories differently than experienced mothers who have established routines and expectations. Third, the 42-day assessment coincides with the end of traditional “zuoyuezi” confinement, potentially inflating the salience of practical assistance as mothers resume normal activities.

Despite these alternatives, the separation of instrumental support into practical assistance and childcare domains aligns with documented changes in Chinese family structures. Research shows urban Chinese fathers spend 2.5 times more hours on childcare than their rural counterparts, but similar time on household tasks. Studies indicate that 68% of Chinese infants receive significant grandparent care, potentially allowing fathers to specialize in specific support types. The transition from one-child to three-child policy has shifted family dynamics, with fathers increasingly expected to participate in direct childcare. These patterns are consistent with qualitative research from other developing countries, where fathers have reported distinct conceptualizations of hands-on infant care versus household assistance as separate support domains (39, 40).

The PBSS-22’s psychometric performance is comparable to of that other breastfeeding-related instruments developed in diverse contexts. For example, maternal breastfeeding self-efficacy measures have consistently demonstrated strong internal consistency and predictive relevance (10), and recent paternal attitude instruments have similarly reported acceptable reliability for assessing partner-related breastfeeding constructs (37). In addition, context-specific breastfeeding support measures, such as workplace-oriented support scales, further underscore that the expression and organization of breastfeeding-related support can vary across settings and cultures (31). The Brazilian Partner Breastfeeding Support Scale identified two factors explaining 52% of the variance (41), while the Persian Breastfeeding Support Scale achieved comparable reliability (*α* = 0.91) but retained the original three-factor structure (42), suggesting that the four-factor solution may be particularly relevant to East Asian family systems. Collectively, these findings support the interpretability of the PBSS-22 as a partner-specific measure that complements broader breastfeeding-related assessments while capturing culturally salient paternal-support behaviors in the early postpartum period.

The PBSS-22’s psychometric properties compare favorably with those of international instruments. The Japanese Breastfeeding Support Scale (J-BSS) reported *α* = 0.83–0.85 across three factors, similar to our dimensional reliabilities (43). The Brazilian Partner Breastfeeding Support Scale found two factors explaining 52% of the variance, less than our 66.5% [23](44). The Malawian Father Involvement Scale identified five factors but with lower reliability (*α* = 0.72–0.81), possibly reflecting greater cultural heterogeneity (45). The Persian Breastfeeding Support Scale achieved comparable reliability (*α* = 0.91) but retained the original three-factor structure, suggesting cultural variations in support conceptualization (42). These comparisons reveal that while core support dimensions appear to be universal, their expression and differentiation vary culturally. The PBSS-22’s four-factor model may reflect China’s unique blend of traditional collectivism and rapid modernization.

Although the PBSS-22 enables domain-specific assessments, evidence for personalized interventions remains preliminary. Recent meta-analytic evidence demonstrates that paternal support interventions can significantly improve exclusive breastfeeding rates at multiple time points: within 1 week postpartum (RR 1.28; 95% CI 1.16, 1.42), 30–42 days postpartum (RR 1.12; 95% CI 1.02, 1.23), and 3 months postpartum (RR 1.35; 95% CI 1.21, 1.50) (2). However, optimal intervention strategies require further empirical validation. Clinicians should use PBSS-22 scores as screening indicators, not diagnostic criteria; consider scores within broader family and cultural contexts; recognize that high scores do not guarantee breastfeeding success given multiple influencing factors; and avoid assuming universal intervention approaches based on dimensional scores.

The PBSS-22 is not suitable for populations with different family structures (e.g., single mothers, same-sex couples), cross-cultural comparisons without further validation, predicting breastfeeding duration without additional clinical factors, assessing support quality versus quantity, or evaluating non-partner support sources. Future research priorities should include test–retest reliability over 2-week intervals, convergent validity with established support measures, and predictive validity for 6-month exclusive breastfeeding. Methodological advances should incorporate dyadic analysis, including paternal perspectives, and ecological momentary assessment of daily support behaviors. Clinical translation requires randomized trials of PBSS-guided interventions, the development of clinical cutoff scores, and integration with electronic health records. Theoretical development should focus on the longitudinal modeling of support trajectories, investigation of support-outcome mediators, and cross-cultural invariance testing.

## 5 Conclusion

This study successfully developed and psychometrically validated the PBSS-22, providing a culturally appropriate, multidimensional instrument for quantifying paternal breastfeeding support in Chinese postpartum care. The scale demonstrated strong internal consistency, robust content validity, and a coherent four-factor structure, enabling the domain-specific identification of support gaps. Practically, the PBSS-22 can be used by maternity hospitals, nurses, and community health services to screen support deficiencies and design family centered counseling that strengthens fathers’ emotional, informational, practical, and childcare support behaviors. Future research should confirm the factor structure using confirmatory factor analysis in an independent multicenter cohort, evaluate measurement invariance across key subgroups (e.g., delivery mode and urban–rural residence), and establish predictive validity for breastfeeding duration and maternal well-being.

## Data Availability

The raw data supporting the conclusions of this article will be made available by the authors, without undue reservation.
